# Concussion: an Introduction

**DOI:** 10.2217/cnc.15.7

**Published:** 2016-03-01

**Authors:** Hannah Wilson

The term concussion is used interchangeably with mild traumatic brain injury (mTBI) and is defined as a complex pathophysiologic process affecting the brain, induced by biomechanical forces secondary to direct or indirect forces to the head [1].

In recent months, much focus has been placed on the long-term effects of repeated concussions on athletes participating in contact sports [2]. The US National Football League (NFL) has come under particular scrutiny following high-profile suicides of former players and the bringing of a lawsuit by retired players and their relatives against the NFL in an effort to secure compensation for the lasting effects of head trauma sustained during professional games [3,4]. Sports-related concussions are not limited to American football, with data from a number of sports including ice hockey, boxing and rugby indicating the prevalence of concussive injuries [5,6].

Sportsmen and women are far from the only group for whom concussions are a health priority. Blast injury has been branded the signature injury for US veterans deployed in the Iraq and Afghanistan wars, with 20% of deployed military personnel suffering a head injury during this time, 83% of which were uncomplicated TBI or concussion (mTBI) [7]. TBI has been identified as a potential primary driver of overall adverse outcomes in this population, further emphasizing our need for increased understanding.

First described as a progressive neurodegeneration observed in boxers in the 1920s, chronic traumatic encephalopathy (CTE) remains a controversial topic. Suggested to result from concussive and subconcussive injuries and clinically associated with symptoms common in old age including memory disturbances, personality changes, Parkinsonism and speech and gait changes; formal diagnosis of CTE remains restricted to post-mortem examination, although there is promise for a number of *in vivo* diagnostic techniques, particularly PET imaging [8,9]. In addition to symptomatic overlaps, CTE shares some features of Alzheimer’s disease, notably tau-immunoreactive neurofibrillary tangles, neuropil neurites and in some cases the presence of diffuse senile plaques [10], and it is hoped that by furthering our understanding of CTE we may also gain insight into the development of Alzheimer’s.

At present, concussion is categorized based on traditional and insensitive methods, which give little insight into disease phenotype and specific pathophysiology. The need for validated biomarkers and objective criteria to enhance the specificity of diagnosis and treatment is paramount [11]. The search for and validation of biomarkers for concussion research remains in early stages but there are a number of promising avenues for exploration. Total tau, S-100 calcium-binding protein B and neuron-specific enolase concentrations are amongst others being investigated as potential blood biomarkers to aid diagnosis and clinical decision-making following concussion [8]. Cognitive function, balance and eye movement-based tests such as the King–Devick test [12], mBESS [13] and that described by Samadani and colleagues [14] provide alternative methods of concussion detection and monitoring.

The need for reliable biomarkers is not limited to diagnosis; monitoring of recovery following concussion is key to successful rehabilitation and such markers may be invaluable in informing return to play decisions, the intricacies of which are discussed by Rose and colleagues [15]. Monitoring blows to the head during play or deployment has also been a focus for the development of novel technologies, with wearable sensors gaining prominence and a number of studies investigating their use commencing in recent months. In particular, it is hoped that combining remote accelerometric data from such wearable sensors with traditional blood and urine sampling may aid in the identification of markers for injury and recovery [16].

The launch of *Concussion* comes as discussion and controversies surrounding the topic are at an all-time high. Through an online, open access format we hope that the journal will provide a timely and freely accessible forum for discussion and debate, bringing together neurologists, sports medics and those on the frontline. Employing double-blind peer review and the guidance of our editorial board, *Concussion* will present an unbiased platform in this often controversial field. As evidenced in our inaugural articles, the journal will publish research, review and opinion pieces from experienced authors discussing all aspects of concussion research, including pathology, diagnosis, management, biomarker discovery and validation, post-concussion syndrome, recovery and rehabilitation, and long-term effects. The journal is supported by an international editorial board of experts in various aspects of concussion research and practice, to whom we are very grateful for all their time and work towards the success of *Concussion*. We would also like to extend our thanks to all the authors and peer reviewers who have contributed thus far.

We hope you enjoy the inaugural content of *Concussion*, I would be delighted to receive any feedback, comments or suggestions on the journal or any of the articles featured.
